# Elevated CSF angiopoietin-2 correlates with blood-brain barrier leakiness and markers of neuronal injury in early Alzheimer’s disease

**DOI:** 10.1038/s41398-023-02706-w

**Published:** 2024-01-05

**Authors:** Carol Van Hulle, Selvi Ince, Ozioma C. Okonkwo, Barbara B. Bendlin, Sterling C. Johnson, Cynthia M. Carlsson, Sanjay Asthana, Seth Love, Kaj Blennow, Henrik Zetterberg, J. Scott Miners

**Affiliations:** 1 Wisconsin Alzheimer’s Disease Research Center, School of Medicine and Public Health, University of Wisconsin-Madison, Madison, WI USA; 2 Department of Medicine, University of Wisconsin School of Medicine and Public Health, Madison, University of Wisconsin-Madison, Madison, WI USA; 3 Dementia Research Group, Clinical Neurosciences, Bristol Medical School, University of Bristol, Bristol, UK; 4 Wisconsin Alzheimer’s Institute, School of Medicine and Public Health, University of Wisconsin-Madison, Madison, WI USA; 5 Geriatric Research Education and Clinical Center, William S. Middleton Memorial Veterans Hospital, Madison, WI USA; 6 Department of Psychiatry and Neurochemistry, Institute of Neuroscience and Physiology, the Sahlgrenska Academy at the University of Gothenburg, Mölndal, Sweden; 7 Clinical Neurochemistry Laboratory, Sahlgrenska University Hospital, Mölndal, Sweden; 8 Department of Neurodegenerative Disease, UCL Institute of Neurology, Queen Square, London, UK; 9 UK Dementia Research Institute at UCL, London, UK; 10 Hong Kong Center for Neurodegenerative Diseases, Clear Water Bay, Hong Kong, China

**Keywords:** Molecular neuroscience, Predictive markers

## Abstract

Breakdown of the neurovascular unit is associated with blood-brain barrier (BBB) leakiness contributing to cognitive decline and disease pathology in the early stages of Alzheimer’s disease (AD). Vascular stability depends on angiopoietin-1 (ANGPT-1) signalling, antagonised by angiopoietin-2 (ANGPT-2) expressed upon endothelial injury. We examined the relationship between CSF ANGPT-2 and CSF markers of BBB leakiness and core AD biomarkers across three independent cohorts: (i) 31 AD patients and 33 healthy controls grouped according to their biomarker profile (i.e., AD cases *t*-tau > 400 pg/mL, *p*-tau > 60 pg/mL and Aβ42 < 550 pg/mL); (ii) 121 participants in the Wisconsin Registry for Alzheimer’s Prevention or Wisconsin Alzheimer’s Disease Research study (84 participants cognitively unimpaired (CU) enriched for a parental history of AD, 20 participants with mild cognitive impairment (MCI), and 17 with AD); (iii) a neurologically normal cohort aged 23–78 years with paired CSF and serum samples. CSF ANGPT-2, sPDGFRβ, albumin and fibrinogen levels were measured by sandwich ELISA. In cohort (i), CSF ANGPT-2 was elevated in AD and correlated with CSF *t*-tau and *p*-tau181 but not Aβ42. ANGPT-2 also correlated positively with CSF sPDGFRβ and fibrinogen – markers of pericyte injury and BBB leakiness. In cohort (ii), CSF ANGPT-2 was highest in MCI and correlated with CSF albumin in the CU and MCI cohorts but not in AD. CSF ANGPT-2 also correlated with CSF *t*-tau and *p*-tau and with markers of neuronal injury (neurogranin and α-synuclein) and neuroinflammation (GFAP and YKL-40). In cohort (iii), CSF ANGPT-2 correlated strongly with the CSF/serum albumin ratio. Serum ANGPT-2 showed non-significant positive associations with CSF ANGPT-2 and the CSF/serum albumin ratio. Together, these data indicate that CSF and possibly serum ANGPT-2 is associated with BBB leakiness in early AD and is closely related to tau pathology and neuronal injury. The utility of serum ANGPT-2 as a biomarker of BBB damage in AD requires further study.

## Introduction

Vascular pathology and cerebral vascular dysfunction are demonstrable in most patients with Alzheimer’s disease (AD), which shares common risk factors with cerebrovascular disease (reviewed [1]). Neurovascular uncoupling and leakiness of the blood–brain barrier (BBB) contribute to cognitive decline and AD pathology (reviewed [2, 3]). In an imaging study of people with pre-clinical AD (i.e., having a clinical dementia rating of 0.5), BBB leakiness within the hippocampus was related to elevated CSF soluble platelet-derived growth factor receptor β (sPDGFRβ), a marker of pericyte injury. We previously reported that CSF sPDGFRβ level was elevated and correlated with CSF *t*-tau and *p*-tau levels in clinical AD patients confirmed by CSF biomarker status (i.e., *t*-tau > 400 pg/mL, *p*-tau > 60 pg/mL and Aβ42 < 550 pg/mL) [4]. CSF sPDGFRβ has since been shown to be positively correlated with CSF *t*-tau and *p*-tau in two independent cohorts of cognitively unimpaired participants with biomarker changes indicative of a transition from normal ageing to early AD [5, 6]. CSF sPDGFRβ also correlated with PET-tau signal, and both markers were inversely related to cerebral blood flow, the associations being stronger in PET Aβ-positive individuals [7]. A recent study reported that sPDGFRβ level was highest in MCI and was elevated in MCI-converters compared to MCI patients whose cognitive performance remained stable over a 1-year period [5]. Together, the data point to microvessel-tau interactions that are associated with BBB leakiness and reduced blood flow, and which are probably exacerbated by earlier deposition of Aβ.

Angiopoietin (ANGPT) signalling via tyrosine kinase with immunoglobulin-like and EGF-like domains 1 and 2 (TIE-1 and TIE-2) receptors on endothelial cells, regulates vascular stability and BBB permeability in adult tissues [8, 9]. ANGPT-1, released by pericytes, activates TIE-2 receptors on endothelial cells, mediating vascular stability and BBB integrity. ANGPT-2, released predominantly by endothelial cells in response to injury, acts as a weak agonist or an antagonist of TIE-2, and is associated with angiogenesis [10, 11] and BBB leakiness via disruption of cadherin and tight junction protein expression [8, 12]. ANGPT-2 is upregulated in response to hypoxia, by a hypoxia-inducible factor (HIF)1α-dependent mechanism [13] and via inflammatory cytokines [14], and can be regulated by VEGF signalling [15]. Circulatory ANGPT-2 levels are raised in multiple cancers and in conditions, such as sepsis, associated with vascular leakage (reviewed in [16]).

Recombinant ANGPT-2 induces BBB leakiness, potentially via endothelial apoptosis, in a cortical cold-injury rat model [12]. Infarct size and BBB permeability after transient middle cerebral artery occlusion in an ANGPT-2 gain-of-function mouse model, were reversed on restoration of Tie-2 signalling [8]. ANGPT-2 levels were raised in the vitreous humour in patients with diabetic retinopathy (DR) [17] and were chronically elevated in a rodent model of DR [18]. In the rodent model, recombinant ANGPT-2 triggered pericyte loss and BBB leakiness [18]. Elevated ANGPT-2 may result from hyperglycaemia-induced pericyte-drop-out and endothelial injury associated with sustained inflammation, leading to BBB leakage in mouse models of DR [19]. ANGPT-2-neutralising antibodies reverse BBB leakiness, by limiting pericyte drop-out and reducing inflammation [20]. ANGPT-2 expression was previously reported to be elevated in microvessel-enriched preparations of brain tissue in AD [21], in which pericyte loss and BBB leakiness have been reported at an early disease stage [22–24]. ANGPT-2 was elevated concurrently with markers of angiogenesis in the cortex of young (2 month) mice in an APP over-expressing J20 mouse model of AD [25]. Together, these studies indicate that ANGPT-2 is raised locally within the brain in disease conditions associated with cerebral vascular injury and BBB leakiness and raise the possibility that such upregulation would occur in the early stages of AD.

In this study, we have explored the relationships between CSF ANGPT-2; markers of pericyte injury (CSF sPDGFRβ) and BBB leakiness (CSF fibrinogen and albumin); established markers of core AD pathology (CSF Aβ and tau); and markers of neuronal injury (neurogranin and α-synuclein) and neuroinflammation (GFAP and YKL-40), in three independent cohorts. The first cohort comprised AD and controls stratified according to CSF AD biomarkers; the second consisted predominantly of at-risk cognitively unimpaired (CU) controls, but also included individuals with mild cognitive impairment (MCI) and patients with established AD. In a third cohort, we investigated the relationships between ANGPT-2 in CSF and serum, and between ANGPT-2 and the CSF/serum albumin ratio, in paired CSF and serum samples from neurologically normal adult donors.

## Methods

### Study cohorts

Cohort (i): CSF aliquots from clinical diagnostic CSF samples from 33 AD cases and 31 controls were kindly provided by the Clinical Neurochemistry Laboratory at Sahlgrenska University Hospital (courtesy of Professor Blennow). CSF *t*-tau, *p*-tau181, and Aβ42 had previously been measured using commercial ELISAs (INNOTEST, Fujirebio, Belgium). Patients whose CSF had abnormal levels of AD biomarkers (*t*-tau > 400 pg/mL, *p*-tau > 60 pg/mL and Aβ42 < 550 pg/mL) were classified as having AD [26]. The demographics of the cohort including gender and age at which CSF was collected, are shown in Table 1a. Cognitive status and *APOE* genotype were not recorded in these individuals. The study complied with Swedish Biobank law (Biobanks in Medical Care Act) and was approved by the Ethical Committee at University of Gothenburg, Sweden.

**Table 1:** a Summary of cohort (i). b Summary of Cohort (ii), *N* = 121. **c** Summary of cohort (iii).

	Cases	Gender	Age at LP	CSF AB42	CSF *t*-tau	CSF *p*-tau
Control	*n* = 31	18 M:12 F	68.4 ± 12.5	819.7 ± 213.8	233.7 ± 77.4	41.2 ± 9.9
AD	*n* = 33	18 M:15 F	76 ± 6.5	424.3 ± 80.6	734.2 ± 228.6	87.0 ± 20.7

	Normal (*N* = 84)	MCI (*N* = 20)	Dementia (*N* = 17)	*p* value
Sex				0.019
Female, *n* (%)	57 (67.9%)	8 (40.0%)	7 (41.2%)	
Male, *n* (%)	27 (32.1%)	12 (60.0%)	10 (58.8%)	
*APOE* ε4 carriership				0.006
* APOE*-, *n* (%)	49 (58.3%)	8 (40.0%)	3 (17.6%)	
* APOE*+, *n* (%)	35 (41.7%)	12 (60.0%)	14 (82.4%)	
Age at LP				< 0.001
Mean (SD)	62.1 (5.83)	69.9 (6.36)	68.8 (5.92)	
Amyloid status				< 0.001
A-, *n* (%)	54 (66.7%)	3 (15.0%)	0 (0.0%)	
A+, *n* (%)	27 (33.3%)	17 (85.0%)	17 (100.0%)	
Tau status				< 0.001
T-, *n* (%)	67 (82.7%)	6 (30.0%)	1 (5.9%)	
T+, *n* (%)	14 (17.3%)	14 (70.0%)	16 (94.1%)	

Cases	Age +/− SD	Gender
***n*** = **23**	**58.1** + **20.3**	**12** **M:11** **F**

Amyloid positivity was defined as CSF Aβ42/40 less than or equal to 0.046. Tau positivity was defined as CSF pTau181 concentration greater than 24.8 pg/mL. Clinical diagnosis was determined through a clinical consensus conference without reference to biomarker data.

*A±* amyloid status, *ANGPT-2* angiopoietin-2, *APOE4* apolipoprotein E ε4, *CU* cognitively unimpaired, *LP* lumbar puncture, *MCI* mild cognitive impairment, *sPDGFRβ* soluble platelet-derived growth factor receptor beta, *T±*  tau status.

Cohort (ii): CSF aliquots were provided from the Wisconsin Registry for Alzheimer’s Prevention Study and Wisconsin Alzheimer’s Disease Research Center (WISC cohort). WISC participants’ cognitive performance and functional status had been adjudicated by consensus conference. Diagnoses of MCI or dementia due to suspected AD were assigned based on National Institute on Aging-Alzheimer’s Association criteria [14, 15], without reference to biomarkers. The WISC sample included donors with a clinical diagnosis of ‘cognitively unimpaired’ (CU; *n* = 84), mild-cognitive impairment (*n* = 20), and established AD dementia (*n* = 17) at baseline. All WISC participants had baseline CSF obtained by lumbar puncture (LP). For this study, CU participants were selected if they had had serial LP sampling of CSF. The demographics, including sex distribution, *APOE* genotype, age at LP, and Aβ and tau status, are summarised in Table 1b. Markers of AD pathology (Aβ42, Aβ40, *t*-tau and *p*-tau181) and a panel of markers of neuronal injury and neuroinflammation had previously been measured using Roche ® robust prototype immunoassays part of the NeuroToolKit (Roche Diagnostics International Ltd, Switzerland) and have been reported in a previous study [27]. Study participants provided consent prior to all study visits. Study procedures were approved by the University of Wisconsin-Madison Institutional Review Board.

Cohort (iii): Paired serum and CSF aliquots from neurologically normal controls (*n* = 23) spanning a wide age-range (21–86 years) were obtained from the Blennow/Zetterberg lab. The CSF/serum albumin ratio had previously been determined by an immunoturbidimetric albumin method (Elecsys, Roche Diagnostics, Penzberg, Germany). The demographics of the cohort, including sex and age at LP, are presented in Table 1c. The study complied with Swedish Biobank law (Biobanks in Medical Care Act) and was approved by the Ethical Committee at the University of Gothenburg, Sweden.

### ANGPT-2 ELISA measurement in CSF and serum

ANGPT-2 level was measured by ELISA (Quantikine kit, R & D systems, U.K.) according to the manufacturer’s instructions. CSF was diluted 2-fold and serum 5-fold in a proprietary dilution buffer. Absorbance was read at 450 nm in a FLUOstar OPTIMA plate reader (BMG labtech, Aylesbury, UK). Measurements were made in a single well for serum and CSF, the concentration of ANGPT-2 was determined by interpolation against a standard curve generated by serially diluting recombinant ANGPT-2 (3000–23.5 pg/ml).

### Albumin ELISA measurement in CSF

CSF albumin level was measured in CSF samples from cohort (ii), by commercial sandwich ELISA (Cat no 108788) (Abcam, Cambridge, UK) as in our previous study [4]. CSF samples were diluted 1 in 2000 and measured in duplicate. Absorbance was read at 450 nm in a FLUOstar OPTIMA plate reader (BMG labtech, Aylesbury, UK) and albumin concentration was interpolated from a standard curve derived by serial dilution of recombinant human albumin (200–3.125 ng/mL). Results are expressed in µg/ml after correction for dilution.

### Fibrinogen ELISA measurement in CSF

Fibrinogen levels were measured in Cohort (i) by a commercially available sandwich ELISA (Cat. no. EH3057, Finetest, Wuhan, China) following the manufacturer’s instructions. In brief, CSF samples diluted 1 in 500 in PBS, were measured in duplicate, and the averages were determined after interpolation against a standard curve derived from serial dilutions of recombinant human fibrinogen (100–1.56 ng/ml).

### sPDGFRβ ELISA measurement in CSF

sPDGFRβ had previously been measured in CSF in Cohort (i) and Cohort (iii) by a commercially available sandwich ELISA (Cat no EHPDGFRB, Thermo Fisher Scientific) following the manufacturer’s instructions. We followed the same protocol to measure sPDGFRβ in CSF samples from cohort (ii). In brief, CSF samples (100 μl undiluted) were measured in duplicate, and the averages determined after interpolation against a standard curve derived from serial dilutions of recombinant human PDGFRβ (18,000–24 pg/mL).

### Statistical analysis

ANGPT-2 datasets were normally distributed. A single outlier was identified in the serum ANGPT-2 measurements and was removed prior to analysis. Pearson’s partial correlation (removing age effects) were calculated for all CSF biomarkers. Linear mixed-effects models with random intercepts, age-at-lumbar-puncture as the measure of time, and CSF vascular biomarker as the outcome were used to test associations with tau positivity (> 24.8 pg/mL), and cognitive status.

## Results

### CSF ANGPT-2 is elevated in AD and correlates with markers of BBB leakiness

**Fig. 1:**
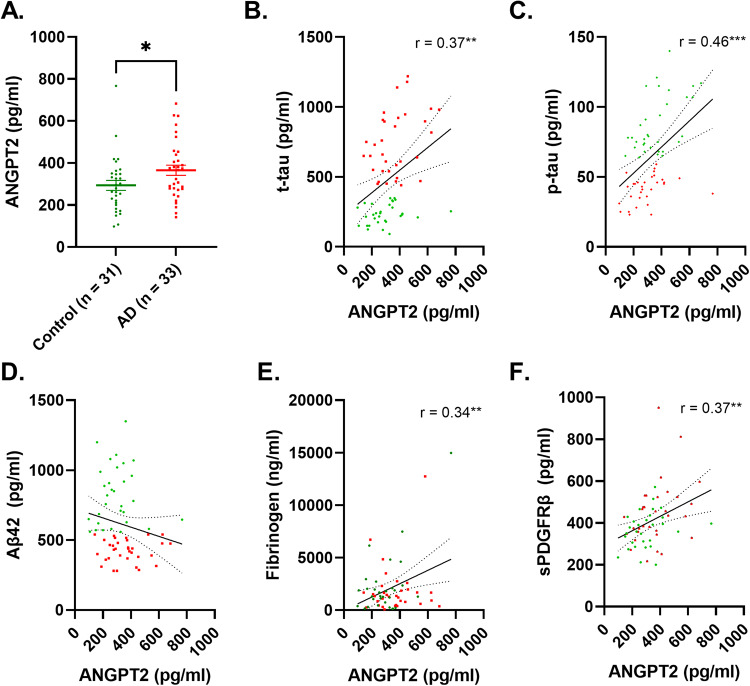
CSF level of ANGPT-2 is elevated in AD in relation to CSF-tau and markers of BBB breakdown in Alzheimer’s disease. **A** Dot plot showing significantly higher levels of ANGPT-2 in Alzheimer’s disease (AD) (*p* = 0.039; unpaired *t*-test). **B–D** Scatterplot showing a positive correlation between ANGPT-2 and *t*-tau and *p*-tau181; no correlation was observed for Aβ42. **E,** **F** Scatterplots showing a positive correlation between CSF ANGPT-2 level and CSF markers of BBB (fibrinogen and sPDGFRβ). In **A** the bars represent the mean±SEM. In **B–F** Pearson correlation coefficient (r) and the best-fit linear regression line is shown and 95% confidence intervals are superimposed. Each dot represents an individual sample. *p* < 0.05 was considered statistically significant.

CSF ANGPT-2 level was significantly higher in AD patients than controls (*p* < 0.05) (Fig. 1A). CSF ANGPT-2 correlated positively with *t*-tau (*r* = 0.37, *p* < 0.01) and more strongly with *p*-tau181 (*r* = 0.46, *p* < 0.001) (Fig. 1B, C) but not with Aβ42 (*r* = −0.18, *p* = 0.15) (Fig. 1D). ANGPT-2 correlated positively with CSF fibrinogen (*r* = 0.34, *p* < 0.01) and CSF sPDGFRβ (*r* = 0.37, *p* < 0.01) (Fig. 1E, F).

CSF ANGPT-2 level did not correlate with age in controls (*r* = 0.11, *p* = 0.55) but correlated strongly with age in AD cases (*r* = 0.58, *p* = 0.0004) in cohort (i) (Supplementary Fig. 1). In cohort (ii), there was a non-significant weak relationship between age and CSF ANGPT-2 level across the AD continuum (*r* = 0.17, *p* = 0.06).

### CSF ANGPT-2 is elevated in MCI and correlates with markers of BBB leakiness, neuronal injury and neuroinflammation

**Fig. 2:**
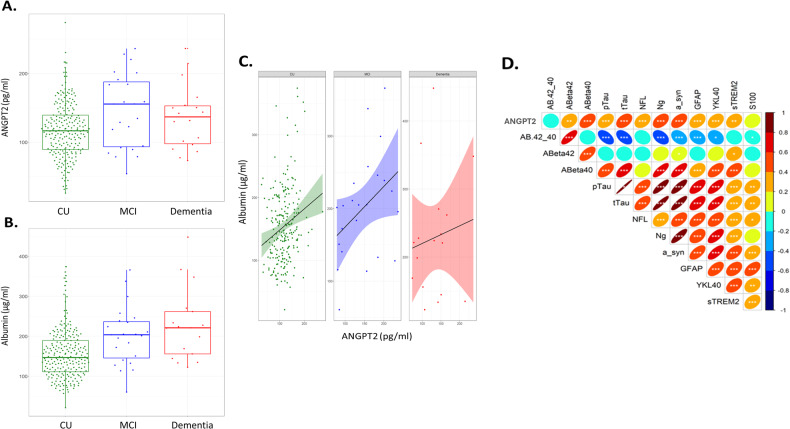
CSF ANGPT-2 level is elevated in MCI and correlates with markers of BBB leakiness, neuronal injury and neuroinflammation. **A** Boxplot showing elevated ANGPT-2 in MCI (*n* = 20) compared to cognitively unimpaired (CU) controls (*n* = 84) (*p* = 0.03). ANGPT-2 level did not differ significantly between AD (*n* = 17) and CU controls (One-way ANOVA). **B** CSF albumin levels are higher in MCI (*p* = 0.068) and significantly higher in AD (*p* = 0.0009) compared to CU controls (One-way ANOVA). **C** CSF ANGPT-2 is positively correlated with CSF albumin level in the CU and MCI groups but not in the AD group (Pearson correlation coefficient (r)). **D** A summary of Pearson’s correlation coefficients between CSF ANGPT-2 and CSF levels of disease pathology (Aβ40, Aβ42, *t*-tau, *p*-tau181); neuronal injury (neurogranin (ng) and alpha-synuclein (a-syn, neurofilament light (nfl) and S100)) and neuroinflammation (GFAP, sTREM-2 and S100). *p < 0.05, **p < 0.01, ***p < 0.001.

CSF ANGPT-2 was highest in MCI subjects and was significantly higher than cognitively unimpaired (CU) controls (*p* = 0.03) (Fig. 2A). Albumin level was raised in MCI, but not significantly (*p* = 0.068), and was significantly higher in AD than CU controls (*p* = 0.0006) (Fig. 2B). Among CU participants, CSF ANGPT-2 but not albumin was associated with tau-positive status, i.e., CSF *p*-Tau181 > 24.8 pg/mL (*p* = 0.05 and p = 0.87 respectively). CSF ANGPT-2 correlated with albumin in the CU (*r* = 0.21, *p* = 0.0008) and MCI groups (*r* = 0.43, *p* = 0.06) but not in AD cases (*r* = 0.09, *p* = 0.57) (Fig. 2C). CSF ANGPT-2 correlated with sPDGFRβ across the entire cohort (*r* = 0.37, *p* = 0.0034).

CSF ANGPT-2 level correlated positively with *t*-tau (*r* = 0.42, *p* < 0.0001) and *p*-tau181 (*r* = 0.39, *p* < 0.0001), and with Aβ40 (*r* = 0.44, *p* < 0.0001) but not Aβ42 (*r* = −0.03, *p* = 0.76). ANGPT-2 correlated with markers of neuronal injury – neurogranin (*r* = 0.43, *p* < 0.0001) and α-synuclein (*r* = 0.46, *p* < 0.0001); and markers of neuroinflammation – GFAP (*r* = 0.36, *p* < 0.0001) and YKL-40 (*r* = 0.34, *p* = 0.0002). A summary of the correlations between ANGPT-2 and markers of AD pathology, neuronal injury and inflammation is shown in Fig. 2D.

### CSF ANGPT-2 correlates with the CSF/serum albumin ratio in matched CSF and serum samples from neurologically normal controls

**Fig. 3:**
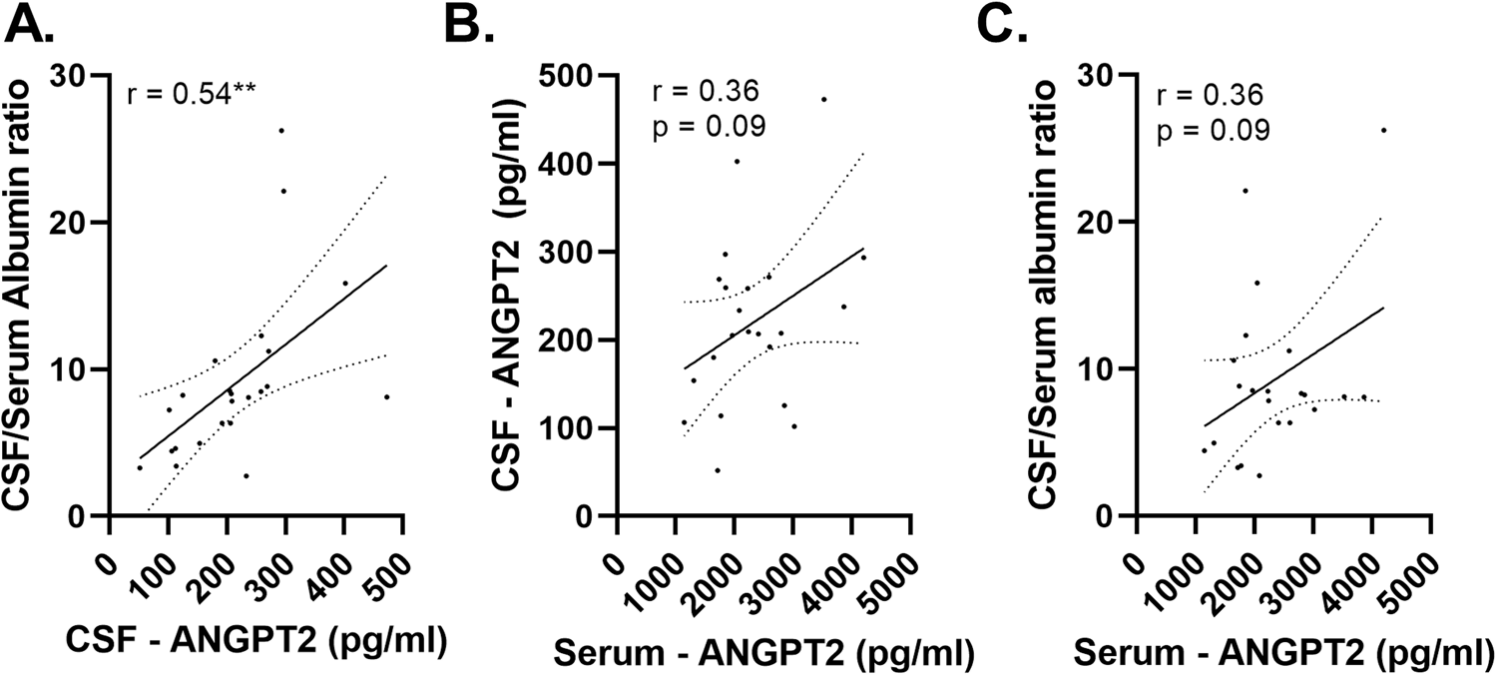
CSF and serum ANGPT-2 correlated with CSF/serum albumin ratio. A scatterplot showing a positive correlation between CSF and serum ANGPT-2 in matched serum and CSF samples. Scatterplots showing positive correlation between CSF and serum ANGPT-2 and the CSF/serum albumin ratio. Pearson correlation coefficient (r) is shown. The best-fit linear regression line is shown and 95% confidence intervals are superimposed. Each dot represents an individual sample. *p* < 0.05 was considered statistically significant. **p < 0.01.

CSF ANGPT-2 level correlated strongly with the CSF/serum albumin ratio in matched CSF and serum samples from neurologically normal individuals (*n* = 23) (*r* = 0.54, *p* < 0.01) (Fig. 3A). Serum ANGPT-2 tended to be associated with CSF ANGPT-2 (*r* = 0.36, *p* = 0.09), and with the CSF/serum albumin ratio (*r* = 0.36, *p* = 0.09) but these relationships were not statistically significant (Fig. 3B, C). Neither CSF nor serum ANGPT-2 correlated with age: despite a trend towards a rise in CSF ANGPT-2 with age, the correlations did not reach statistical significance (*p* = 0.40, *r* = 0.06) (Supplementary Fig. 1)

## Discussion

In this study, we show elevated CSF ANGPT-2 levels in individuals with CSF biomarker positivity for AD, based on established cut-off values for *t*-tau, *p*-tau181 and Aβ42 [26], which correlated with CSF *t*-tau and *p*-tau181, and with markers of pericyte injury (sPDGFRβ) and BBB leakiness (CSF fibrinogen). Elevation of ANGPT-2 is likely to occur early in the development of disease - in an independent cohort spanning the full spectrum of cognitive decline in AD, CSF ANGPT-2 was highest in MCI and correlated most strongly with CSF albumin level in the cognitively unimpaired (CU) and MCI groups compared to AD, despite CSF albumin levels continuing to rise with disease progression. Across the same cohort, ANGPT-2 correlated positively with CSF sPDGFRβ and with CSF *t*-tau and *p*-tau181 and correlated with CSF markers of neuroinflammation (YKL-40, GFAP and sTREM2) and neuronal injury (neurogranin and α-synuclein). In a third cohort comprising matched serum and CSF samples from healthy controls, CSF ANGPT-2 correlated with the CSF/serum albumin ratio, a marker of BBB leakiness. Serum ANGPT-2 showed a trend towards positive correlation with both CSF ANGPT-2 and the CSF/serum albumin ratio. Together, these data indicate that CSF ANGPT-2 is a potential marker of BBB leakiness, and associated with tau pathology and neuronal injury in the early stages of AD. Whether, like CSF ANGPT-2, serum ANGPT-2 proves to be a useful indicator of BBB integrity will need to be determined in a larger study.

The ANGPT-TIE signalling pathway is a key regulator of vascular stability and is dysregulated in diseases including stroke and diabetic retinopathy, in which elevated ANGPT-2 is associated with BBB leakiness and endothelial apoptosis [12, 28]. Whether the ANGPT-TIE pathway is disrupted in AD is less well understood. ANGPT-2 level was reported to be elevated in microvessels enriched from post-mortem AD brain tissue compared to healthy age-matched controls [21]. ANGPT-2 expression was elevated in young 2-month-old APP over-expressing J20 mice at an age when markers of pathological angiogenesis and increased vessel density were also observed suggesting that ANGPT-2 contributes to vascular instability in the stages of AD prior to the onset of disease pathology [25]. A clinical study in the Knight-ADRC (*n* = 311) and ADNI (*N* = 293) cohorts previously showed that CSF ANGPT-2 level was strongly associated with the ratio of ptau181:Aβ42 – a predictor of conversion from unimpaired cognition to very mild/mild AD [29].

In this study, CSF ANGPT-2 level was highest in MCI patients and correlated with CSF albumin and sPDGFRβ, a marker of pericyte injury that was previously shown to be elevated in early AD (clinical dementia rating 0.5) and to correlate with MRI evidence of BBB breakdown within the hippocampus [22, 23]. CSF ANGPT-2 level was strongly related to CSF *t*-tau and *p*-tau181 but not Aβ42, as also reported for sPDGFRβ [4–7]. Data from the above Knight-ADRC/ADNI study also showed that CSF ANGPT-2 levels were associated with *p*-tau181 and not Aβ42. A recent study revealed that CSF sPDGFRβ level correlated positively with the CSF/serum albumin ratio, a marker of BBB leakiness, in patients with clinical dementia rating between 0 and 0.5 but not in established AD [5]; the CSF/serum albumin ratio rose steadily with disease progression, as we found for CSF albumin in the present study. Mediation analysis revealed that BBB breakdown in the CDR 0–0.5 group reflected the indirect influence of Aβ on pericyte degeneration, whilst BBB damage at a later disease stage was a direct effect of Aβ, by then presumably more abundant [5]. CSF sPDGFRβ was also shown to increase in normal ageing, after adjustment for vascular risk factors and *APOE*, and closely followed age-related changes in CSF *t*- and *p*-tau across 303 cognitively unimpaired individuals from the Chinese Alzheimer’s Biomarker and Lifestyle study (CABLE) [6]. In the same study, CSF sPDGFRβ levels were further increased in a separate pre-clinical AD cohort, independent of ageing, and were strongly related to tau changes. Here, CSF and serum ANGPT-2 levels did not increase significantly with normal ageing but was strongly related to age in AD. In the larger cohort (ii), changes in CSF ANGPT-2 were independent of normal ageing. Taken together, the parallels between ANGPT-2 and sPDGFRβ suggest that abnormal ANGPT-TIE signalling is related to normal ageing but is exacerbated, likely due to enhanced pericyte degeneration and BBB leakiness, in the early stages of AD coinciding with pathological changes in tau. Whether the initial accumulation of Aβ accelerates this process, as has been shown for sPDGFRβ [7] remains to be determined.

CSF markers of neuroinflammation and endothelial injury (ICAM-1, VCAM-1, YKL-40, IL-15 and VEGF-A) were reported to be elevated in the pre-clinical stages of AD and to be tightly associated with CSF tau, markers of cognitive decline, and cortical thinning – the relationship was strongest in individuals who were Aβ-positive on PET scan [30]. In our study, CSF ANGPT-2 level was strongly associated with CSF YKL-40 and GFAP, general markers of neuroinflammation, associated with astrogliosis in neurodegenerative conditions [31, 32]. ANGPT-2 was also moderately associated with CSF sTREM-2, which was previously reported to be elevated in MCI and strongly related to *t*-tau and *p*-tau181 but not Aβ42 [33]. The authors of this last study suggested that the rise in CSF sTREM-2 reflected microglial activation in response to neuronal degeneration. We also found a strong correlation between CSF ANGPT-2 and two additional CSF markers of neurodegeneration: neurogranin and α-synuclein. CSF neurogranin was reported to be elevated in AD in association with increased *t*-tau and *p*-tau [34], and to be strongly related to cognitive decline [35]. CSF α-synuclein was also found to be raised in early AD and to be related to cognitive decline [36]. ANGPT-2 was recently shown to be elevated in cortical neurones in young 2-month old J20 mice with evidence of pathological angiogenesis and was induced in response to Aβ peptides in neural stem cells suggesting that Aβ-induced ANGPT-2 expression within neurons contributes to vascular instability in early AD [25]. Together, these data highlight the linkage between neuroinflammation, cerebral vascular injury, and neurodegeneration in AD.

CSF ANGPT-2 levels do not correlate with CSF Aβ42; however, they are strongly correlated with CSF Aβ40. We and others previously reported a similar pattern of correlations for sPDGFRβ [4–6]. CSF Aβ40 level is reduced in cerebral amyloid angiopathy [37], probably as a consequence of increased Aβ40 accumulation in cerebral blood vessels. The relationship between CSF ANGPT-2 and Aβ40 suggests that CAA severity (not assessed in this study) may have an impact on the expression of these vascular injury markers in CSF in AD. Integrity of the BBB, which is compromised in CAA, is important for the clearance of toxic peptides from the brain. Pericytes internalise and clear Aβ peptides via LRP-1 mediated phagocytosis [38] and LRP-1 mediates the transcytosis of Aβ across the endothelium [39]. The number of NG2-positive pericytes within the hippocampus was inversely related to the amount of guanidine-extracted insoluble Aβ40 (but not Aβ42) load [40] in human post-mortem brain tissue. Of probable relevance is the finding that fibrillar Aβ40 is toxic to pericytes in culture [40]. The accumulation of Aβ40, particularly in CAA, may contribute to BBB leakiness and pericyte damage in AD.

We previously reported that serum and CSF sPDGFRβ levels correlated positively in paired serum and CSF samples from healthy donors, but that serum sPDGFRβ was not related to the CSF/serum albumin ratio [4]. In the present study, serum ANGPT-2 tended to be associated with CSF ANGPT-2 and the CSF/serum albumin ratio, although neither correlation reached statistical significance. Serum ANGTP1 level was previously been shown to be higher in AD than controls [41]. The utility of serum ANGPT-2 (or ANGPT-1) as an indicator of BBB function will need to be determined through larger studies, preferably in those that also include MCI and AD patients. The origin of angiopoietin peptides within the CNS is unclear, they may be produced locally from injured blood vessels within the brain; however, the close relationship between ANGPT-2 CSF and serum levels may also reflect leakage into the brain in conditions associated with a leaky BBB, such as AD. Serum ANGPT-2 is elevated in multiple cancers and diseases associated with microvasculature dysfunction. Its usefulness is therefore unlikely to be as a marker of AD per se; its correlation with several other AD-related biomarkers is likely to reflect the importance of vascular dysfunction and BBB breakdown in the development and progression of the disease, and its utility is likely to be greatest in combination with other disease-specific markers, as a means of detecting and monitoring BBB integrity.

If the ANGPT-TIE signalling pathway is deregulated in the early stages of AD, as suggested by our findings, this pathway would be a promising target for therapeutic intervention. The TIE-2 receptor agonist AV-001, which opposes the effects of ANGPT-2, was shown to restore cognition in a rat model of multiple microinfarcts [42]. Vaculotid, a TIE-2 agonist, also accelerated recovery following experimentally induced stroke in a rat model of diabetes [43]. In a Phase II clinical trial, the bispecific antibody faricimab, a dual inhibitor of ANGPT-2 and VEGF, improved visual acuity and reduced central subfield thickness in diabetic macular oedema [44].

In conclusion, CSF ANGPT-2 appears to be a sensitive marker of pericyte injury and BBB breakdown in early AD. Its rise is closely related to tau pathology and neuronal degeneration, and also to neuroinflammation. Future studies in longitudinal cohorts, combining clinical assessment, CSF and serum analysis with high-resolution MRI, will inform on the timing of cerebral vascular function in relation to cognitive decline and the onset and regional spread of disease pathology in AD. The utility of serum ANGPT-2 as a marker of pericyte injury and BBB breakdown merits further investigation.

### Supplementary information

Supplementary Figure 1

## Data Availability

N/A

## References

[1] Love S, Miners JS (2016). Cerebrovascular disease in ageing and Alzheimer’s disease. Acta Neuropathol.

[2] Sweeney MD, Montagne A, Sagare AP, Nation DA, Schneider LS, Chui HC (2019). Vascular dysfunction-the disregarded partner of Alzheimer’s disease. Alzheimers Dement.

[3] Sweeney MD, Sagare AP, Zlokovic BV (2018). Blood-brain barrier breakdown in Alzheimer disease and other neurodegenerative disorders. Nat Rev Neurol.

[4] Miners JS, Kehoe PG, Love S, Zetterberg H, Blennow K (2019). CSF evidence of pericyte damage in Alzheimer’s disease is associated with markers of blood-brain barrier dysfunction and disease pathology. Alzheimers Res Ther.

[5] Lv X, Zhang M, Cheng Z, Wang Q, Wang P, Xie Q (2023). Changes in CSF sPDGFRbeta level and their association with blood-brain barrier breakdown in Alzheimer’s disease with or without small cerebrovascular lesions. Alzheimers Res Ther.

[6] Wang J, Fan DY, Li HY, He CY, Shen YY, Zeng GH (2022). Dynamic changes of CSF sPDGFRbeta during ageing and AD progression and associations with CSF ATN biomarkers. Mol Neurodegener.

[7] Albrecht D, Isenberg AL, Stradford J, Monreal T, Sagare A, Pachicano M (2020). Associations between vascular function and tau PET are associated with global cognition and amyloid. J Neurosci.

[8] Gurnik S, Devraj K, Macas J, Yamaji M, Starke J, Scholz A (2016). Angiopoietin-2-induced blood-brain barrier compromise and increased stroke size are rescued by VE-PTP-dependent restoration of Tie2 signaling. Acta Neuropathol.

[9] Hayashi SI, Rakugi H, Morishita R (2020). Insight into the role of angiopoietins in ageing-associated diseases. Cells.

[10] Lv LL, Du YT, Chen X, Lei Y, Sun FY. Neuroprotective effect of angiopoietin2 is associated with angiogenesis in mouse brain following ischemic stroke. Brain Sci. 2022;12(11):1428. 10.3390/brainsci12111428 PMC9688484 36358355

[11] Pichiule P, LaManna JC (2002). Angiopoietin-2 and rat brain capillary remodeling during adaptation and deadaptation to prolonged mild hypoxia. J Appl Physiol.

[12] Nag S, Papneja T, Venugopalan R, Stewart DJ (2005). Increased angiopoietin2 expression is associated with endothelial apoptosis and blood-brain barrier breakdown. Lab Invest.

[13] Mandriota SJ, Pyke C, Di Sanza C, Quinodoz P, Pittet B, Pepper MS (2000). Hypoxia-inducible angiopoietin-2 expression is mimicked by iodonium compounds and occurs in the rat brain and skin in response to systemic hypoxia and tissue ischemia. Am J Pathol.

[14] Mandriota SJ, Pepper MS (1998). Regulation of angiopoietin-2 mRNA levels in bovine microvascular endothelial cells by cytokines and hypoxia. Circ Res.

[15] Lobov IB, Brooks PC, Lang RA (2002). Angiopoietin-2 displays VEGF-dependent modulation of capillary structure and endothelial cell survival in vivo. Proc Natl Acad Sci USA.

[16] Saharinen P, Eklund L, Alitalo K (2017). Therapeutic targeting of the angiopoietin-TIE pathway. Nat Rev Drug Discov.

[17] Tsai T, Alwees M, Asaad MA, Theile J, Kakkassery V, Dick HB (2023). Increased angiopoietin-1 and -2 levels in human vitreous are associated with proliferative diabetic retinopathy. PLoS ONE.

[18] Hammes HP, Lin J, Wagner P, Feng Y, Vom Hagen F, Krzizok T (2004). Angiopoietin-2 causes pericyte dropout in the normal retina: evidence for involvement in diabetic retinopathy. Diabetes.

[19] Tuuminen R, Haukka J, Loukovaara S (2015). Poor glycemic control associates with high intravitreal angiopoietin-2 levels in patients with diabetic retinopathy. Acta Ophthalmol.

[20] Ogura S, Kurata K, Hattori Y, Takase H, Ishiguro-Oonuma T, Hwang Y (2017). Sustained inflammation after pericyte depletion induces irreversible blood-retina barrier breakdown. JCI Insight.

[21] Thirumangalakudi L, Samany PG, Owoso A, Wiskar B, Grammas P (2006). Angiogenic proteins are expressed by brain blood vessels in Alzheimer’s disease. J Alzheimers Dis.

[22] Montagne A, Barnes SR, Sweeney MD, Halliday MR, Sagare AP, Zhao Z (2015). Blood-brain barrier breakdown in the aging human hippocampus. Neuron.

[23] Nation DA, Sweeney MD, Montagne A, Sagare AP, D’Orazio LM, Pachicano M (2019). Blood-brain barrier breakdown is an early biomarker of human cognitive dysfunction. Nat Med.

[24] Miners JS, Schulz I, Love S (2018). Differing associations between Abeta accumulation, hypoperfusion, blood-brain barrier dysfunction and loss of PDGFRB pericyte marker in the precuneus and parietal white matter in Alzheimer’s disease. J Cereb Blood Flow Metab.

[25] Sheikh AM, Yano S, Tabassum S, Mitaki S, Michikawa M, Nagai A (2023). Alzheimer’s amyloid beta peptide induces angiogenesis in an alzheimer’s disease model mouse through placental growth factor and angiopoietin 2 expressions. Int J Mol Sci.

[26] Hansson O, Zetterberg H, Buchhave P, Londos E, Blennow K, Minthon L (2006). Association between CSF biomarkers and incipient Alzheimer’s disease in patients with mild cognitive impairment: a follow-up study. Lancet Neurol.

[27] Van Hulle C, Jonaitis EM, Betthauser TJ, Batrla R, Wild N, Kollmorgen G (2021). An examination of a novel multipanel of CSF biomarkers in the Alzheimer’s disease clinical and pathological continuum. Alzheimers Dement.

[28] Akwii RG, Sajib MS, Zahra FT, Mikelis CM (2019). Role of angiopoietin-2 in vascular physiology and pathophysiology. Cells.

[29] Harari O, Cruchaga C, Kauwe JS, Ainscough BJ, Bales K, Pickering EH (2014). Phosphorylated tau-Abeta42 ratio as a continuous trait for biomarker discovery for early-stage Alzheimer’s disease in multiplex immunoassay panels of cerebrospinal fluid. Biol Psychiatry.

[30] Janelidze S, Mattsson N, Stomrud E, Lindberg O, Palmqvist S, Zetterberg H (2018). CSF biomarkers of neuroinflammation and cerebrovascular dysfunction in early Alzheimer disease. Neurology.

[31] Llorens F, Thune K, Tahir W, Kanata E, Diaz-Lucena D, Xanthopoulos K (2017). YKL-40 in the brain and cerebrospinal fluid of neurodegenerative dementias. Mol Neurodegener.

[32] Abu-Rumeileh S, Steinacker P, Polischi B, Mammana A, Bartoletti-Stella A, Oeckl P (2019). CSF biomarkers of neuroinflammation in distinct forms and subtypes of neurodegenerative dementia. Alzheimers Res Ther.

[33] Suarez-Calvet M, Morenas-Rodriguez E, Kleinberger G, Schlepckow K, Araque Caballero MA, Franzmeier N (2019). Early increase of CSF sTREM2 in Alzheimer’s disease is associated with tau related-neurodegeneration but not with amyloid-beta pathology. Mol Neurodegener.

[34] Wellington H, Paterson RW, Portelius E, Tornqvist U, Magdalinou N, Fox NC (2016). Increased CSF neurogranin concentration is specific to Alzheimer disease. Neurology.

[35] Liu W, Lin H, He X, Chen L, Dai Y, Jia W (2020). Neurogranin as a cognitive biomarker in cerebrospinal fluid and blood exosomes for Alzheimer’s disease and mild cognitive impairment. Transl Psychiatry.

[36] Twohig D, Rodriguez-Vieitez E, Sando SB, Berge G, Lauridsen C, Moller I (2018). The relevance of cerebrospinal fluid alpha-synuclein levels to sporadic and familial Alzheimer’s disease. Acta Neuropathol Commun.

[37] Verbeek MM, Kremer BP, Rikkert MO, Van Domburg PH, Skehan ME, Greenberg SM (2009). Cerebrospinal fluid amyloid beta(40) is decreased in cerebral amyloid angiopathy. Ann Neurol.

[38] Ma Q, Zhao Z, Sagare AP, Wu Y, Wang M, Owens NC (2018). Blood-brain barrier-associated pericytes internalize and clear aggregated amyloid-beta42 by LRP1-dependent apolipoprotein E isoform-specific mechanism. Mol Neurodegener.

[39] Storck SE, Meister S, Nahrath J, Meissner JN, Schubert N, Di Spiezio A (2016). Endothelial LRP1 transports amyloid-beta(1–42) across the blood-brain barrier. J Clin Invest.

[40] Schultz N, Brannstrom K, Byman E, Moussaud S, Nielsen HM, Netherlands Brain B (2018). Amyloid-beta 1–40 is associated with alterations in NG2+ pericyte population ex vivo and in vitro. Aging Cell.

[41] Schreitmuller B, Leyhe T, Stransky E, Kohler N, Laske C (2012). Elevated angiopoietin-1 serum levels in patients with Alzheimer’s disease. Int J Alzheimers Dis.

[42] Culmone L, Powell B, Landschoot-Ward J, Zacharek A, Gao H, Findeis EL (2022). Treatment with an angiopoietin-1 mimetic peptide improves cognitive outcome in rats with vascular dementia. Front Cell Neurosci.

[43] Venkat P, Ning R, Zacharek A, Culmone L, Liang L, Landschoot-Ward J (2021). Treatment with an angiopoietin-1 mimetic peptide promotes neurological recovery after stroke in diabetic rats. CNS Neurosci Ther.

[44] Sahni J, Patel SS, Dugel PU, Khanani AM, Jhaveri CD, Wykoff CC (2019). Simultaneous inhibition of angiopoietin-2 and vascular endothelial growth factor-a with faricimab in diabetic macular edema: BOULEVARD phase 2 randomized trial. Ophthalmology.

