# Supplementary material for: Elevated CSF angiopoietin-2 correlates with blood-brain barrier leakiness and markers of neuronal injury in early Alzheimer’s disease
Source: Transl Psychiatry. 2024 Jan 5;14:3. doi: 10.1038/s41398-023-02706-w (PMC10770135; doi:10.1038/s41398-023-02706-w)
Supplement: Supplementary file 1 — Supplementary Figure 1 [file 41398_2023_2706_MOESM1_ESM.docx]

**Supplementary Figure 1**


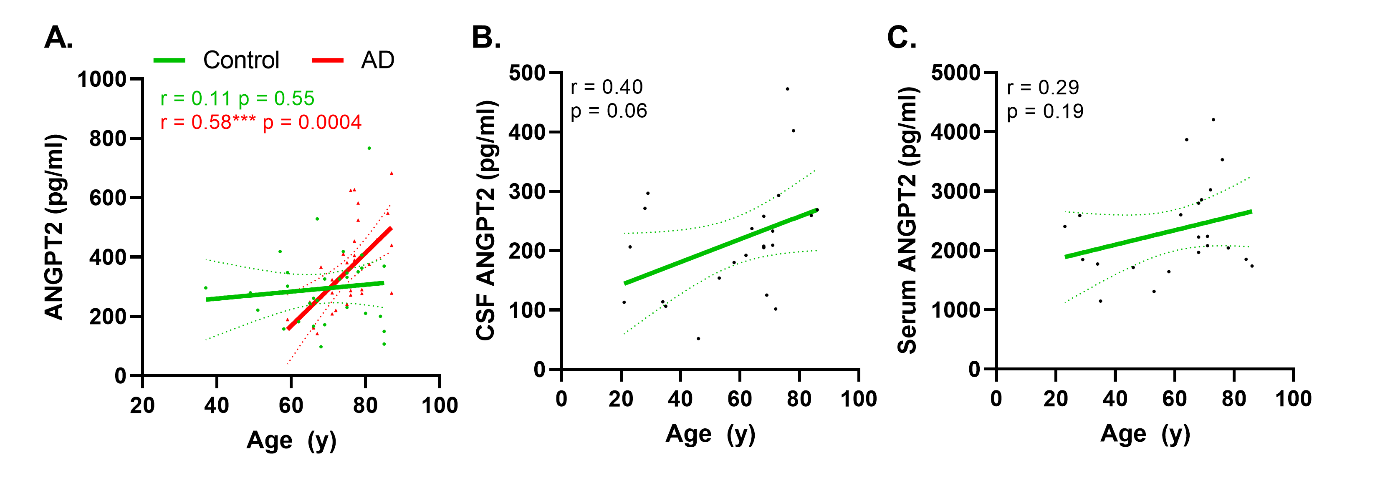


Supplementary Figure 1. CSF ANGPT2 levels are elevated in relation to age in Alzheimer’s disease. (A) Scatterplot showing the relationship with age in AD (n = 33) and age-matched controls (n = 31) from cohort (i). (B-C) Scatterplots showing relationship between age and CSF and serum ANGPT2 in cohort (iii) from neurologically normal controls (n = 23) spanning 23-84 years. The best-fit linear regression line is shown and 95% confidence intervals are superimposed. Each dot represents an individual sample. p < 0.05 was considered statistically significant. *** p < 0.001
